# Hepatic Disorders and COVID-19: From Pathophysiology to Treatment Strategy

**DOI:** 10.1155/2022/4291758

**Published:** 2022-12-08

**Authors:** Parisa Shiri Aghbash, Hamed Ebrahimzadeh Leylabadlo, Hamidreza Fathi, Mohaddeseh Bahmani, Rojin Chegini, Hossein Bannazadeh Baghi

**Affiliations:** ^1^Immunology Research Center, Tabriz University of Medical Sciences, Tabriz, Iran; ^2^Department of Virology, Faculty of Medicine, Tabriz University of Medical Sciences, Tabriz, Iran; ^3^Liver and Gastrointestinal Diseases Research Center, Tabriz University of Medical Sciences, Tabriz, Iran; ^4^Network of Immunity in Infection, Malignancy and Autoimmunity (NIIMA), Universal Scientific Education and Research Network (USERN), Tabriz, Iran; ^5^Drug Applied Research Centre, Tabriz University of Medical Sciences, Tabriz, Iran; ^6^Metabolic Liver Disease Research Center, Isfahan University of Medical Sciences, Isfahan, Iran; ^7^Infectious and Tropical Diseases Research Center, Tabriz University of Medical Sciences, Tabriz, Iran

## Abstract

Following the SARS-CoV-2 outbreak and the subsequent development of the COVID-19 pandemic, organs such as the lungs, kidneys, liver, heart, and brain have been identified as priority organs. Liver diseases are considered a risk factor for high mortality from the COVID-19 pandemic. Besides, liver damage has been demonstrated in a substantial proportion of patients with COVID-19, especially those with severe clinical symptoms. Furthermore, antiviral medications, immunosuppressive drugs after liver transplantation, pre-existing hepatic diseases, and chronic liver diseases such as cirrhosis have also been implicated in SARS-CoV-2-induced liver injury. As a result, some precautions have been taken to prevent, monitor the virus, and avoid immunocompromised and susceptible individuals, such as liver and kidney transplant recipients, from being infected with SARS-CoV-2, thereby avoiding an increase in mortality. The purpose of this review was to examine the impairment caused by SARS-CoV-2 infection and the impact of drugs used during the pandemic on the mortality range and therefore the possibility of preventive measures in patients with liver disease.

## 1. Introduction

In December 2019, a pneumonia outbreak started in Wuhan, Hubei Province, China. Its causative agent was then identified as a previously unknown coronavirus and was given the interim name novel coronavirus 2019 (2019-nCoV). In February 2020, the World Health Organization (WHO), based on taxonomy and phylogeny, renamed 2019-nCoV as severe acute respiratory syndrome coronavirus-2 (SARS-CoV-2), while the resulting disease was designated coronavirus disease 2019 (COVID-19). SARS-CoV-2 is a positive-sense single-stranded RNA virus with a length of 27–32 KB from the subfamily *Orthocoronavirinae* [1]. The virus can be transmitted from person to person through respiratory droplets and close contact. Parallels have been noted in the occurrence of SARS-CoV-2 and SARS-CoV. The clinical manifestations of COVID-19 include fever, dry cough, and diarrhea [2]. In addition, liver dysfunction has also been reported in studies [1]. Thus, autopsies performed on fatal severe acute respiratory syndrome cases have said that fat degradation and central lobule necrosis of liver tissue have been seen in patients who have died through severe acute respiratory syndrome (SARS) [3]. SARS-CoV was identified in the liver of infected individuals, and about 60% of patients with SARS experienced symptoms of liver injury [4]. Besides, an increase in liver enzymes was reported in patients with the Middle East respiratory syndrome-related coronavirus (MERS-CoV) infection in 2012, along with records of peri-venular necrosis and mild portal inflammation [5]. As a consequence, during infection, SARS-CoV-2 can be contained in human liver tissue. The prevalence of liver damage in SARS-CoV-2 virus infection is 14–53% and is recurrent but mild [6]. It is also assumed that drugs used for SARS-CoV-2 treatment such as lopinavir/ritonavir, remdesivir, chloroquine, and tocilizumab have destructive and toxic effects on the liver [7]. However, despite the significant prevalence of steatosis and hepatic fibrosis reported in autopsies, liver injury in COVID-19 is subclinical [8]. A study by Fan et al. showed that liver disorders are more common in men [2]. Besides, another study found that the angiotensin-converting enzyme 2 (ACE2) receptor is significantly increased in women's liver tissue, implicating a better prognosis compared to men [9]. COVID-19-associated liver damage is defined as any liver injury during the course and treatment of COVID-19 in patients with or without underlying liver diseases [10]. Also, liver damage may be due to direct or indirect effects of SARS-CoV-2, such as septic shock, ischemia, multiorgan dysfunction, drug-related toxicity, and hepatitis due to the inflammatory response of the immune system following the cytokine storm due to the evolution of COVID-19 [10]. This study aimed to understand the mechanisms and pathways that play a role in the development of liver disease caused by SARS-CoV-2, to reduce the incidence of clinical symptoms and complications following COVID-19 recovery.

## 2. Mechanisms of Liver Injury in COVID-19

Liver injury following SARS-CoV-2 infection or drug usage manifests as microvascular steatosis and mild lobular and portal activity, according to the liver autopsy findings in COVID-19 deceased patients [11]. Furthermore, studies have shown that ACE2, like SARS-CoV, acts as a receptor for SARS-CoV-2, which is expressed in more than 80% of alveolar cells in the lungs [12, 13]. Other organs, in addition to the lungs, have been reported to express ACE2; as a result, they are directly affected by the cytopathic effects of SARS-CoV-2 [14]. ACE2 has also been significantly expressed in the gastrointestinal tract, vascular endothelium, and cholangiocytes [12]. Besides, transmembrane protease serine 2 (TMPRSS2), the co-receptor for SARS-CoV-2 needed to release viral particles, is expressed in cholangiocytes and liver cells [15]. In this regard, SARS-CoV-2 binds to this receptor and enters the target cell, leading to damage to cholangiocytes and liver cells [15]. On the other hand, viral infection leads to cholangiocyte susceptibility to hepatic disorders caused by SARS-CoV-2 through dysfunction of genes involved in the formation of tight junctions and bile acid transmission [16].

**Figure 1:**
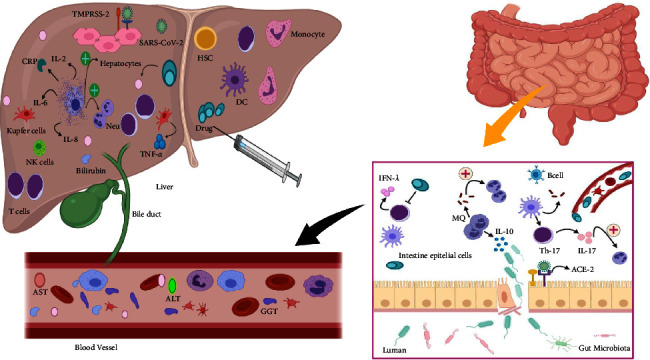
SARS-CoV-2 function in liver disorder. The SARS-CoV-2 virus can either bind directly to its receptors on the surface of liver cells, such as ACE2 and TMPRSS2, or it can be indirectly through the effect of antiviral drugs or transmitted through the gastrointestinal tract and exert its influence. Besides, increased intrinsic immune system activity such as macrophage cells and the production of proinflammatory cytokines and infection-induced lymphopenia exacerbates clinical symptoms and liver disorders.

Nonetheless, the exact mechanisms associated with these disorders are debatable. However, some of the assumed mechanisms include the following:

1. Increased immune response activity following SARS-CoV-2 infection: intrinsic immune activity in the liver plays an essential role during COVID-19. In this regard, the number of liver macrophages significantly increases following the disease. Thus, the production of proinflammatory cytokines and biomarkers such as c-reactive protein (CRP), serum ferritin, lactate dehydrogenase (LDH), D-dimer, interleukin (IL)-6, IL-2, and excessive activity of cytotoxic T cells following infection with SARS-CoV-2 and consequent liver tissue damage has also been reported [17, 18].
2. Active replication of the virus in hepatocytes and cholangiocytes, which in turn leads to cytotoxicity: pathobiological studies indicate the presence of SARS-CoV-2 RNA in liver cells [19]. As mentioned, ACE2 is expressed in hepatocytes and bile duct epithelial cells, and as a result, SARS-CoV-2 binds to target cells via ACE2. Therefore, the liver serves as a potential target for direct viral infection. In this regard, following the targeting of bile ducts and liver cells, damage to these areas leads to liver cirrhosis and sepsis [20]. Moreover, during biopsy specimens of COVID-19 victims, it was found that the cytotoxic effect of the virus on the liver leads to moderate microvascular steatosis and mild lobular and portal activity [7].
3. Gut-liver axis: as mentioned above, ACE2 is highly expressed in small intestinal cells. The virus may also be transmitted through the reticular system of the liver due to venous blood circulation from the small intestine to the liver [6]. Also, due to the detection of viral RNA in fecal samples, the virus can be transmitted by potential circulation from the intestine to the liver [10].
4. Anoxia: respiratory failure is one of the complications of COVID-19, which in turn leads to hypoxic hepatitis following severe hypoxia [21].
5. Drug-induced liver injury (DILI): it has been reported that antiviral drugs can lead to liver disorders and damage. In this regard, we can mention lopinavir/ritonavir, remdesivir, chloroquine, tocilizumab, and uminefovir [21].
6. Reactivation of pre-existing liver disease: studies show that patients with chronic liver diseases are more prone to liver disorders and damage following infection with the SARS-CoV-2 virus. Besides, drugs such as tocilizumab and baricitinib lead to hepatitis B virus (HBV) reactivation and eventual liver dysfunction. The effect of SARS-CoV-2 infection on cholestasis exacerbation in cholestatic liver patients is also debatable (Figure 1).

## 3. Immune Response and Inflammation in Liver Injury

Inflammatory cytokine storms increase the secretion of inflammatory cytokines by the over-activity of lymphocytes and macrophages [22]. In this regard, it was observed that the activity of the innate and acquired immune systems following infection with SARS-CoV-2 is associated with systemic inflammatory response syndrome (SIRS) [23]. Dysfunction of the innate immune response may be one of the causes of liver disorders following SARS-CoV-2 infections. It has been shown that changes in the levels of biomarkers such as CRP, lymphocytes, neutrophils, and cytokines such as IL-6 can, in turn, lead to liver disorders [24]. Moreover, cytokine storm, also known as cytokine release syndrome (CRS), identified CRP, IL-6, LDH, and ferritin promotion, leading to ARDS and liver disorders [25]. It has also been reported that the progression of SARS-CoV-2 disease is due to the systemic production of proinflammatory cytokines. It is worth noting that during hepatitis, IL-6 is a potent cytokine, and compared to other cytokines, such as IL-1*β* and TNF-*α*, it leads to more severe inflammation. This cytokine is also a marker of systemic inflammation due to its half-life [26, 27]. Moreover, it has also been observed that, in people with high disease severity, peripheral blood cell counts such as Th17 and CD8 T, as well as cytokines such as IL-2, IL-6, IL-7, IL-10, G-CSF, MCP-1, and TNF-*α*, ratio increases in people with lower disease severity [28–30]. Moreover, IFN secretion in COVID-19 is directly related to hepatic impairment and retinoid toxicity; as a result, IFN secreted during infection leads to impaired immune function and toxic effects on oxidative metabolism and mitochondrial activity [20, 31].

Furthermore, the SARS-CoV-2 virus has been reported to trigger several proinflammatory signals through TLRs and activation of CTL cells [32]. Inflammatory signals in COVID-19 are also amplified by TLRs expressed by infected cells. Given the increasing progression of infectious diseases following a decrease in T lymphocytes, inflammatory pathways are activated, which in turn causes macrophage activity and secondary inflammatory reactions. In addition to impairing lung function, these inflammatory responses can impair the function of other organs, such as the liver [29].

Accordingly, in the early stages of infection, the control of liver disorders leads to an inhibition of disease progression. In this regard, liver damage is stimulated by the inflammation of the liver, activating innate immune cells and releasing cytokines. In addition, factors such as CRP 20 mg/ml and lymphopenia are directly related to liver damage. Thus, in 63% to 70.3% of patients with SARS-CoV-2, lymphopenia increases mortality [24].

## 4. The Hypothesis in the Pathogenesis of COVID-19's Liver Damage

Another cause of inflammation in COVID-19-induced liver damage is high concentrations of retinoic acid or retinol esters. In this regard, one of the suggested pathogenesis clues in liver damage is the accumulation of more than 80% of vitamin A in the star cells of the liver. Its excessive increase, in turn, leads to the activity and hypertrophy of liver cells and finally causes fibrosis and liver damage [20]. Evidence suggests that acute hypervitaminosis A is associated with viral hepatitis, with elevated serum and liver concentrations of vitamin A and decreased serum retinol-binding protein 4 (RBP4) [33]. On the other hand, viral infections such as SARS-CoV-2 have been shown to increase the retinoid cascade activity, hepatic apoptotic stimulation by cellular induction, and ultimately impair transient cholestatic liver function [20]. During this disorder, the damaged bile ducts and liver cells secrete stored compounds of vitamin A into the bloodstream. As a result, following increased apoptosis, necrosis, and acute neutrophil infiltration, lungs and other organs are damaged during COVID-19 infection [20]. In this regard, it has been speculated that one of the causes of COVID-19 disease symptoms is retinoid toxicity, which is directly related to the severity of the disease with the serum concentration of vitamin A compounds (retinol esters and retinoic acid metabolites) [20].

Furthermore, ischemic gangrenous cholecystitis is one of the late consequences brought on by the SARS-CoV-2 infection [34]. It develops as a result of host immune response dysfunction, activation of the coagulation cascade, and subsequent medium artery thrombosis [34]. Additionally, it has been demonstrated by histology and immunohistochemical investigations that gallbladder vasculitis, as well as endothelial overexpression of medium-sized arteries, increased CD4 T cells, and excessively elevated macrophage frequencies, contributes to gallbladder endothelial dysfunction [35, 36]. The pathophysiological processes are controversial despite several studies in this area. For instance, it is believed that after infection by SARS-CoV-2, systemic inflammation or immunosuppression directly or indirectly causes late symptoms of cholecystitis through an opportunistic infection [37]. Subclinical coagulopathy of COVID-19 is still associated with gallbladder wall ischemia and small vessel thrombosis. Since less than 1% of gastrointestinal and liver cells express ACE2, it is speculated that modulation of gut microbiota following SARS-CoV-2 infection is in turn effective in liver infection [34]. In fact, adjusting the gut microbiota is a suitable and safe therapeutic approach to prevent disorders caused by the disease COVID-19 [34]. In this regard, Odun-Ayo and Reddy have reported that the administration of probiotics in the SARS-CoV-2 infection leads to a normal balance in the intestinal microbiota and thus reduces the possibility of opportunistic and bacterial infections during the COVID-19 disease [38]. In other words, the administration of various antibiotics, antivirals, and antifungals, as well as systemic corticosteroids, leads to hepatotoxic effects, which can be considered one of the possible components in the pathophysiological process of liver damage in COVID-19 patients [39, 40].

## 5. Chronic Liver Disease

COVID-19 can lead to liver damage; approximately 2 to 11% of patients have chronic liver disease (CLD). In this way, CLD includes nonalcoholic fatty liver disease (NAFLD), alcohol-related disease, and chronic viral hepatitis [41]. Patients with CLD had significant levels of aspartate aminotransferase (AST), alanine aminotransferase (ALT), and alkaline phosphatase (ALP) at the time of admission [41]. However, elevated levels of the hepatic enzymes AST and ALT by various factors are not different in patients with or without CLD [40]. As mentioned earlier, these factors include hepatotoxicity of the drug (anti-inflammatory and antiviral drugs used during hospitalization), proinflammatory cytokines of the immune system, ischemia, and congestion associated with positive pressure ventilation, which in turn lead to liver disorders [41]. Besides, the duration of hospitalization, the length of stay in the intensive care unit (ICU), and the need for ventilation have been reported to be longer in CLD patients than in patients without CLD [41]. Liver cirrhosis and hepatitis B are the most common causes of CLD among COVID-19 patients, with a prevalence of approximately 4%. On the other hand, it was found that the risk of mortality is significantly higher in patients with liver diseases, especially cirrhosis [42]. Cirrhosis-related immune system dysfunction has been reported in patients with advanced chronic liver disease predisposed to SARS-CoV-2 infection [43].

### 5.1. Cirrhosis and COVID-19

As a result of cirrhosis-related immune disorders, severe acute respiratory syndrome from SARS-CoV-2 increased in patients with liver cirrhosis during this outbreak, resulting in severe COVID-19 and increased liver disorder injury [44]. Furthermore, acute or chronic liver dysfunction in individuals with uncompensated liver cirrhosis may occur due to stress or sepsis [45]. Also, cirrhotic patients are more susceptible to the influenza virus than non-cirrhotic patients. However, cirrhotic and COVID-19 patients have a higher risk of acute-on-chronic liver failure (ACLF) [46]. Studies in New York also show that 0.4% of the patients have cirrhosis [47]. As noted, cirrhosis leads to increased mortality in patients with the acute respiratory distress syndrome (ARDS), while the effect of cirrhosis on COVID-19 is controversial. However, it has been shown that patients with COVID-19 with pre-existing liver disease require particular clinical intervention due to impaired immune function [44]. In this regard, monitoring compensated cirrhosis patients and caring for severe patients are challenges during the SARS-CoV-2 epidemic [43].

### 5.2. Hepatitis B Virus (HBV) and COVID-19

Experiments suggest that the severity of COVID-19 may not be affected by chronic infection with HBV, but there are differences of opinion [48]. Besides, the suppression of immune system activity may be effective in severe clinical symptoms and pulmonary disorders due to an overactive immune system [11]. It is hypothesized that severe symptoms and diseases are induced by overactive macrophages and the cytokine cascade, resulting in the dysfunction of several organs. There is also evidence of temporary increases in liver transaminase enzyme levels following systemic viral infections. The increase in the level of transaminases indicates the activity of the immune system and the inflammation caused by the cytokine cascade produced, which leads to “bystander hepatitis” without liver dysfunction [43]. COVID-19 patients with HBV infection have been shown to have an increased risk of liver injuries and diseases, as well as mortality [44]. An analysis of 15 patients with chronic hepatitis B infection and COVID-19 showed a substantial rise in total bilirubin levels and a higher mortality rate than COVID-19 patients without HBV infection. Additionally, Chen et al. found that people with chronic hepatitis B were more likely to develop COVID-19 [49]. Other studies have found no association between the severity of COVID-19 disease and chronic viral hepatitis infection [17].

### 5.3. Hepatocellular Carcinoma (HCC) and COVID-19

The data about the severity of COVID-19 in HCC patients have not been recorded. COVID-19 care in these cases has worse effects than in patients without cancer [19]. According to the AASLD, HCC is a chronic liver disorder that can take up to two months to recover completely. Treatments for HCC, on the other hand, should not be postponed [12]. In this regard, according to a study conducted on 1590 COVID-19 patients with cancer, it was demonstrated that the severity of the disease is higher in cancer patients. These patients are also more susceptible to getting infected with SARS-CoV-2 [50]. In this way, since HCC is cancer, treatment for people with HCC should be delayed [12]. Zhang et al. discovered that in a sample of 28 patients, those with malignancies such as HCC had significantly lower clinical rates than those without malignancies [51]. It has also been reported that the immune system is impaired in these patients due to anemia, hypoproteinemia, and adverse nutritional conditions; it eventually exacerbates the infection caused by SARS-CoV-2 [52]. However, information on HCC and COVID-19 diseases is limited and debatable.

## 6. Pre-existing Liver Diseases

In COVID-19 patients, the prevalence of pre-existing liver disease is 2–11% [44]. It has been reported that elderly patients and those undergoing medical treatment are at greater risk of disease progression and consequent liver damage caused by SARS-CoV-2 [43]. Besides, a meta-analysis by Oyelade et al. demonstrated that patients with pre-existing liver disorders were more susceptible to severe COVID-19 infections and higher mortality [53]. This result may be associated with thrombocytopenia, lymphopenia, elevated alanine aminotransferase (ALT) levels, and hypoalbuminemia [54]. It has been reported that in pre-existing liver conditions, elevated liver enzymes can be a clinical challenge for the patient [55]. Singh and Khan compared the test results of people without liver disease and those of people with pre-existing liver diseases. Experiments indicate the effect of obesity as one of the factors in the progression of COVID-19, as it causes NAFLD or metabolic-associated fatty liver disease (MAFLD) [41]. In this regard, reports have shown that patients with MAFLD also have NAFLD [56].

On the other hand, NAFLD refers to a group of disorders that include nonalcoholic fatty liver disease, the nonprogressive subtype of NAFLD, and the nonalcoholic steatohepatitis type (NASH) [57]. NASH is a potentially progressive form of NAFLD that leads to severe fibrosis and cirrhosis and mortality from liver disease; however, NASH, in addition to steatohepatitis, causes other problems [58]. One of the main pathological features of NASH is ballooned hepatocytes [57]. In addition, Eslam et al. have shown that, due to the low association of NAFLD with metabolic risk factors, NAFLD progresses to MAFLD following excessive alcohol consumption [59]. Furthermore, pre-existing disorders such as MAFLD and NAFLD are expected to increase the production of proinflammatory cytokines, enhance reactive oxygen secretion in COVID-19, and subsequently lead to an increase in inflammatory cytokine cascades [60, 61].

### 6.1. Metabolic-Associated Fatty Liver Disease (MAFLD) and COVID-19

Metabolic-associated fatty liver disease (MAFLD) is another cause of chronic liver disease and affects almost a quarter of the world's population. Besides, MAFLD is considered one of the major metabolic disorders and has pre-existing liver diseases [62]. Little is known about COVID-19 patients who also have MAFLD. There have been reports of COVID-19 progression in people with MAFLD, including liver disorder development during hospitalization and increased viral shedding time[56]. Patients with MAFLD also have a high body mass index (BMI), hypertension, diabetes, and dyslipidemia, which contribute to the disease's progression [56]. In this regard, in a study of 214 Chinese patients, it was observed that after adjustment for age, sex, smoking, diabetes, hypertension, and hyperlipidemia, the presence of MAFLD and obesity was associated with increased severity of COVID-19 infection [63]. COVID-19 severity is greater in MAFLD patients with severe fibrosis than in patients with moderate or mild fibrosis [56]. Because MAFLD patients have no clinical symptoms, they are not considered in the early stages of liver disorders and COVID-19 [56]. The study by Biquard et al. showed that in MAFLD patients, the number of receptors sensitive to the SARS-CoV-2 virus, such as ACE2, is not associated with TMPRSS2 [56]. Phosphatidylinositol 3-phosphate5-kinase (PIKfyve), as well as mRNA expression of the genes related to SARS-CoV-2 infectivity, did not increase significantly [56]. As a result, liver damage due to MAFLD is not associated with increased ACE2 expression. However, MAFLD leads to increased toll-like receptor (TLRs) expression at the level of hepatocytes [64]. In MAFLD and obesity, adipocytes and kupffer cells have been shown to increase the secretion of proinflammatory cytokines such as tumor necrosis factor-alpha (TNF-*α*) [56]. In addition to obesity and diabetes, the insulin resistance of adipose tissue and free fatty acid flux to the liver has been shown to lead to the activation of liver macrophages during MAFLD [56]. *M*1 and *M*2 macrophages are functionally distinct macrophages in the liver. In this respect, macrophage *M*1 has inflammatory activity while macrophage *M*2 has anti-inflammatory activity by increasing chemokine expression [56].

As a consequence, the equilibrium of action of these two cells dictates the patient's clinical state. Researchers conclude that, in MAFLD patients, innate immune system dysfunction contributes to increased pathogenesis. As a result, disease progression is aided by the conversion of inflammation-inducing *M*1 macrophages to inflammation-suppressive *M*2 macrophages [56]. Also, the effect of MAFLD on the progression of COVID-19 is determined by the rate of secretion of proinflammatory mediators such as TNF-*α* and IL-6 [65]. The innate immune cells such as macrophages, natural killer, and natural killer T cells are significantly present in liver tissue. Experiments have shown a chronic rise in insulin levels in MAFLD patients, regardless of whether or not they have diabetes, leading to decreased lung capacity in COVID-19 patients [56].

Moreover, immune deficiency, hepatic metabolic disorders, and systemic abnormalities triggered by MAFLD reverse the role of antiviral responses during SARS-CoV-2 infection [66]. As a result, overweight/obesity, type 2 diabetes, evidence of metabolic dysfunction, chronic lung disease, inflammatory bowel disease (IBD), hypertension, immunodeficiency, and kidney failure are all diagnostic criteria for MAFLD as risk factors, in addition to hepatic steatosis [56, 67]. MAFLD is also considered one of the hepatic indications of metabolic syndrome in COVID-19 patients, with persistent inflammation and association with the cytokine cascade during SARS-CoV-2 infection, both contributing to disease development and death [56].

### 6.2. Nonalcoholic Fatty Liver Disease (NAFLD) and COVID-19

In people with NAFLD, the progression of COVID-19 and the rate of virus shedding are considerably higher. Patients with persistent liver abnormalities have been shown to have NAFLD and a high BMI [6]. A study of 202 patients in China reported that risk factors such as obesity and NAFLD increased the progression of COVID-19 and consequently impaired liver function [6]. Furthermore, data show that NAFLD has a significantly greater impact on disease severity and the occurrence of pulmonary complications, respiratory dysfunction, hypoxemia, progressive systemic inflammatory reaction, and ARDS in COVID-19 patients than in obesity [56].

### 6.3. Nonalcoholic Steatohepatitis (NASH) and COVID-19

Studies have shown that obesity and high BMI in SARS-CoV-2 infection, in turn, exacerbated COVID-19 [52]. There is also a direct link between obesity and the need for mechanical ventilation [68]. Since adipose tissue has more surface receptors than lung tissue, adipose tissue is more susceptible to infection with SARS-CoV-2 [69]. It has been reported that during COVID-19, after adipose tissue becomes infected, the disease then spreads to other organs [70]. Obese people are also more likely to develop NAFLD, leading to severe COVID-19, impaired liver function during hospitalization, and increased virus shedding time [52]. COVID-19 progression is also accelerated in NAFLD patients with comorbidities such as diabetes and hypertension. The development and intensity of COVID-19 also increased in young adults with NAFLD [71]. The bipolar state of macrophages in NAFLD patients has been shown to affect the inflammatory response or host resistance to signals from the gut-intestinal axis during COVID-19. As noted, the progression and severity of COVID-19 disease in patients with NAFLD are due to an imbalance between *M*1 macrophages that promote inflammation and *M*2 macrophages that suppress inflammation [45]. Also, cytokines produced during NAFLD significantly increase the progression of COVID-19. As a result, bone involvement is increased following increased serum levels of monocyte-absorbed chemotherapy (MCP-1) protein in COVID-19 patients. NAFLD progresses to NASH as a result of long-term COVID-19 infection [72].

## 7. Liver Transplantation (LT) and COVID-19

During the SARS-CoV-2 outbreak, transplant recipients are more vulnerable to infection, disease prevalence, and long-term shedding of the infectious virus. With a majority of around 3.7 million individuals, liver transplantation is the world's second most successful solid organ transplant [73]. Depending on the SARS-CoV-2 duration, longevity extension in tissues, and the virus duration in the blood, this virus can also spread from the donor to the organ [74]. However, details about how it spreads from the donor to the recipient have not yet been elucidated. Furthermore, owing to a dysfunctional immune system, studies indicate that transplant patients are more vulnerable to COVID-19 than ordinary people. On the other hand, these people have a lower death rate than ordinary people [52].

Acute liver disease is one of the disorders of liver transplantation in this respect. Acetaminophen toxicity, acute viral hepatitis, antiviral drug-induced liver injury, autoimmune hepatitis, Wilson's disease, acute ischemic hepatitis (shock liver), and acute fatty liver during pregnancy are some of the causes of acute liver failure [75].

Knowledge concerning past coronaviruses, including MERS-CoV and SARS-CoV, shows the primary process of virus replication and the secondary phase of virus clearance upon immune response in the case of SARS-CoV-2 infection [76]. The secondary step can reduce CD4+ T cell counts, CD8+ T cell function, and macrophages, leading to a cytokine cascade and subsequent COVID-19 exacerbation [76]. While immune system regulators modulate the immune response, it is linked to increased viral load and better disease outcomes. As a result, immunomodulation controls the severity of COVID-19 in people with LT [76].

The oversecretion of proinflammatory cytokines such as IL-6, IL-8, and TNF-*α* during COVID-19 causes pulmonary dysfunction, which exacerbates clinical symptoms and is improved by suppressing the immune system [77]. Nevertheless, immunosuppression caused by SARS-CoV-2 infection is being investigated [12]. The immune system is a double-edged sword for COVID-19 status suppression. As a result of the elevated viral load and the resulting prolongation of the disease, the immune response is unnecessarily inhibited [78]. It has also been documented that immunosuppression, after interacting with immune system overexpression, causes an increase in the severity of COVID-19 in transplant recipients; as a result, these individuals have long-term viral shedding with a large viral load, and they spread the virus to other people [79].

According to studies, overuse of antiviral medications, immunosuppressive drugs, and multidrug therapy has been associated with liver transplantation (LT). Furthermore, despite the detailed literature on antiviral therapy during SARS-CoV-2 infection, drug interactions in vulnerable populations, such as transplant recipients, should be considered [75]. As a result, the mortality rate of liver transplant patients is believed to be higher.

In SARS-CoV-2 positive individuals, steroid medications are also used following transplantation to prevent adrenal insufficiency [45]. Also, when co-administering drugs like hydroxychloroquine or azithromycin with calcineurin inhibitors (CNI) and mammalian target of rapamycin (mTOR) inhibitors, CNI and mTOR levels should be controlled [45]. Studies have shown that calcineurin inhibitors (tacrolimus or cyclosporine), mTOR inhibitors (everolimus), and mycophenolate are prescribed to most transplant recipients [76]. In this respect, the findings suggest that calcineurin and mTOR inhibitors had appropriate effects on the severity of COVID-19 disease at regular doses. The use of mycophenolate enhanced the severity of COVID-19 disease, the functions of which are summarized in Table 1.

The direct impact of SARS-CoV-2 on lymphocytes such as CD8+ has been shown to induce lymphopenia, which contributes to disease exacerbation [76]. Chloroquine and hydroxychloroquine have both been found to have antiviral and immunomodulatory properties. Ampicillin, amlodipine, azithromycin, propranolol, and antacids are among the medicines that interfere with it. Tacrolimus levels have been found to be significantly elevated by chloroquine [75]. In this respect, preventive steps should be taken due to the immune suppression status and susceptibility of patients with progressive liver disease and patients after LT to COVID-19 [85]. The unacceptably high mortality rate (20.5%) in patients undergoing surgery during the COVID-19 epidemic, the need to reserve ICU beds, and the increased risk of infection with the SARS-CoV-2 during drug treatment with immune inhibitors are examples of the reasons for the reduction of liver transplantation during the outbreak of the SARS-CoV-2 [19].

Finally, it has been shown that inhibiting immune function causes sustained viral shedding and progression of COVID-19 development in liver transplant recipients [75].

## 8. Hepatic Ischemia and Hypoxic and COVID-19

Yang et al. reported in 2019 that hypoxia caused hepatocyte apoptosis and infiltration of inflammatory cells in both *in vitro* and *in vivo* models of hepatic ischemia and hypoxia. Furthermore, in patients with SARS-CoV-2, ischemia, and hypoxia of tissues and organs are frequent pathophysiological occurrences following COVID-19 [86]. Increased viral replication, cytokine production, inflammation, intravascular coagulation, and hypoxic pulmonary vasoconstriction, all of which are pathophysiological hallmarks of SARS-CoV-2 illness development, might be consequences of increasing hypoxia [87]. Besides, hypoxic hepatitis can result from hepatic ischemia and hypoxia in severe instances. Ischemic liver damage is caused by decreased blood flow to the liver and hypotension; according to research by Chew et al. in 2021, as a consequence, the use of vasopressors was suggested as an alternative for ischemic liver injury [88]. Furthermore, it has been observed that intubation of patients on vasopressors increases the risk of ischemia damage owing to hypoxia [88].

## 9. Drug-Induced Liver Injury

Many medications can affect liver function and, as a result, cause liver damage. Liver disorders have been confirmed to be drug-dependent or drug-independent [52]. The elevation of the liver enzymes asymptomatically and the onset of acute hepatitis are consequences. Examples include antibiotics, anti-inflammatory, antiviral, hepatotoxic antiviral drugs, and steroid medications [26]. As previously mentioned, drug-dependent liver diseases are characterized by moderate microvascular steatosis and mild hepatitis [11]. Also, there was evidence of significant vascular steatosis slight lobular and portal function in liver biopsy specimens following COVID-19 infection or drug utilization [11].

Furthermore, Cai et al. observed that more than 10% of admitted patients had elevated levels of liver enzymes due to the use of prescription medications [89]. However, there is no reliable information on drug-induced liver abnormalities during COVID-19. In this regard, it has been stated that the use of angiotensin II receptor blockers and ACE inhibitors during COVID-19 can cause liver failure [89]. Drug-induced liver injury has also been recorded in people with HCV and the human immunodeficiency virus (HIV) [72]. Consequently, it is believed that liver complications in COVID-19 patients may be related to the usage of possibly hepatotoxic antiviral drugs and antibiotics for bacterial infections [7, 72]. Furthermore, serum levels of the MCP-1 protein have been shown to rise in COVID-19 patients, resulting in severe steatohepatitis [72]. As a result, one of the reasons for the association between COVID-19 and DILI is using steatosis-inducing medications, including sodium valproate, amiodarone, tamoxifen, and methotrexate, as predisposing factors for osteoporosis [72]. For example, tamoxifen, as an estrogen antagonist, has been reported in hepatocellular carriers through hepatotoxicity mechanisms such as reduced fatty acid oxidation beta and steatohepatitis, a risk factor for women 50–70 years of age with mastectomy, diabetes, hysterectomy, high cholesterol, high blood pressure, and osteoporosis [90]. On the other hand, clinical symptoms of steatohepatitis, fibrosis, liver cell necrosis, micronedolar cirrhosis, hepatomegaly, ALT, nausea, vomiting, weakness, and inflammatory infiltration have been reported in cases treated with tamoxifen [90]. Moreover, according to DiGiovanna et al. followed by retinoid therapies, bone demineralization happens in humans due to an excessive soar in the retinoid circulation, which can lead to an excessive soar in the retinoid circulation osteoporosis stimulation [91]. Also, Anthony Mawson et al. reported that the liver damage caused by SARS-CoV-2 infection causes the secretion of retinoic acid and retinol esters stored in liver cells to the circulation in dangerous concentrations without attaching to the protein. The elevation of retinoid level in the bloodstream also causes inflammation in injured tissues like the lungs, heart, blood vessels, and skin [20]. Given the COVID-19 disease pandemic, several anticancer and antiviral drugs have been suggested to prevent and mitigate clinical symptoms and pulmonary insufficiency. These include hydroxychloroquine, azithromycin, lopinavir/ritonavir in combination with or indirectly with interferon-beta, remdesivir, baricitinib, imatinib, darunavir, and mifenovir [45].

As a result, during the COVID-19 pandemic, the Réseau d'Étude Francophone de l'Hépatotoxicité des Produits de Santé (REFHEPS) community reviewed medications related to liver disorders, some of which are mentioned as follows (Table 2):

1. According to LiverTox, remdesivir has not been attributed to liver injury. Besides, in clinical trials using remdesivir, there have been no reports of medication usage and hepatotoxicity [53]. However, the effect of remdesivir on liver damage is debatable, owing to its use in COVID-19 patients and the liver diseases caused by this condition [101]. In this way, hepatic dysfunction has been identified in COVID-19 patients after receiving remdesivir, possibly due to an association with P-glycoprotein (P-GP) inhibitors [93]. A US study of 12 patients with COVID-19 found that liver enzymes increased following remdesivir as clinical signs worsened [45]. Also, a case series study conducted by Grein et al. in 2020 on the effect of remdesivir reported that, despite an escalation in liver enzymes in 23% of patients with COVID-19, clinical improvement was shown in 68% of patients. It can be concluded that remdesivir, despite its minor side effects, can also improve SARS-CoV-2 cases [102].
2. Methylprednisolone has been related to the modulation of cytokine cascades. However, patients with liver cirrhosis are more likely to experience spontaneous bacterial peritonitis and elevate the likelihood of HBV reactivation [52]. Also, according to a study by Jeronimo et al., 0.5 mg/kg/day of methylprednisolone was significantly more common in COVID-19 patients with a moderate to severe status than with placebo or standard care. Moreover, higher doses such as 40 mg of methylprednisolone are recommended in extreme cases [103]. However, the treatment results must be interpreted with caution [103, 104].
3. As previously mentioned, dysfunction develops in many organs during COVID-19 disease due to a cytokine cascade and the subsequent inflammation, including lung, heart, and liver disorders. In COVID-19 patients, immune inhibitors like tocilizumab are used to alleviate excessive inflammation for this purpose. Tocilizumab has been linked to severe liver injury, including acute liver disease, acute hepatitis, and liver transplantation in some cases [93]. Furthermore, using the humanized monoclonal antibody tocilizumab as a form of the IL-6 receptor blocker causes an increase in ALT enzyme levels. Jaundice is an unusual sign of liver injury caused by this medication [26]. Tocilizumab has also been observed to induce a slight rise in LFTs in clinical trials; however, this increase is generally temporary and disappears within 2–6 weeks [24].
4. According to studies, the use of hydroxyl chloroquine has been linked to a minority of cases of DILI [99]. It has established the potential for suppressing viral multiplication by performing clinical trials (>20) on chloroquine in ten hospitals across China following success in vitro trials [24]. In COVID-19, colchicine is used to relieve inflammation caused by the disease. DILI is not caused by the use of this drug in low doses [93].
5. Another drug used during COVID-19 is azithromycin. It is a macrolide molecule that has anti-inflammatory and immune-regulating effects. It also plays a role in many respiratory and infectious diseases by affecting the innate and acquired immune systems [105]. In clinical trials, Gyselinck et al. observed that, by administering a dose of 5 mg/5 days, azithromycin increased the utilization of immune cells such as monocytes; in contrast, by increasing the amount by 1 g, they illustrated an increase in side effects and cardiac toxicity [105, 106].
6. Azithromycin has been confirmed to induce acute liver injury within a few days of beginning therapy and clinical symptoms of cholestatic hepatitis within 1–3 weeks of starting therapy [45]. In conjunction with hydroxyl chloroquine, azithromycin is used as a macrolide antibiotic. The liver damage caused by this drug was hepatocellular, and most patients recovered entirely [99].
7. Interferon-beta has been found to affect asymptomatic and minor liver diseases in research. One of the manifestations of liver diseases affected by it is a slight increase in serum aminotransferase level and a natural and temporary increase in serum alkaline phosphatase level [99].
8. It has been found that patients receiving lopinavir/ritonavir during COVID-19 have a high proportion of elevated liver enzymes (56.1% vs. 25%). Lopinavir/ritonavir has been identified as an HIV treatment approved for coronavirus diseases such as MERS-CoV [12]. However, the utility of these medications in the treatment of COVID-19 is still being investigated. During SARS-CoV-2 infection, lopinavir also serves as a protease inhibitor [12]. Evidence shows that when these two drugs are administered together, the endoplasmic reticulum stress pathway in the liver is stimulated. As a result, the caspase mechanism is activated, which leads to liver cell apoptosis.
9. Additionally, oxidative stress and inflammatory responses occur due to the drug's acceleration of liver injury [12]. According to clinical trials conducted by Cao et al. in 2021, lopinavir-ritonavir did not have adequate inhibitory effects in patients with severe disease compared with standard health care. Also, it has side effects such as gastrointestinal disorders, which include anorexia, nausea, abdominal discomfort, or diarrhea [107].
10. Experiments have shown that lopinavir induces mild to extreme promotion in serum aminotransferase levels, ranging from 3% to 10%. Serum enzyme levels that are elevated differ from hepatocellular to cholestatic [99].
11. It has been proposed that low doses of ritonavir do not raise serum levels of liver enzymes and that any elevated liver enzymes are self-limiting and asymptomatic. Furthermore, because of its enzymatic inhibitory properties, ritonavir causes high plasma levels of concomitant medications, which raises liver disorders [99]. Ritonavir increased lopinavir levels by inhibiting the liver enzyme cytochrome P450 (CYP450). Consequently, cytochrome P450 3A4 (CPY3A4) produces a toxic mediator of ritonavir or other metabolized factors, which causes liver disorders [93].
12. Baricitinib is an inhibitor of Janus kinase (JAK)-1 and 2 approved for treating rheumatoid arthritis. Consequently, its use causes a minor and temporary elevation in transaminase enzyme levels [99]. Based on clinical trials in 2020, the FDA approved the use of baricitinib in combination with remdesivir to treat COVID-19 patients who require invasive mechanical ventilation of excess oxygen or extracellular membrane oxygenation (ECMO) [108]. However, baricitinib alone has not been approved by the FDA to treat COVID-19 [108].
13. During the flu epidemic, oseltamivir was recommended by the WHO for people at high risk for infection [107]. During experiments, oseltamivir was administered to 89.9% of COVID-19 patients in Wuhan, China. However, there is not enough detail about its effect on liver function [12]. Generally, in a study of 1099 patients with SARS-CoV-2, no specific information was obtained regarding the impact of oseltamivir on the treatment and prevention of the SARS-CoV-2 virus [107].
14. Ribavirin can be prescribed as a treatment for SARS-CoV-2 because of its broad spectrum. However, significant toxicities, such as severe hemolysis, have been identified following the use of this drug during this outbreak [12].
15. Studies have also attributed angiotensin-converting enzyme inhibitors (ACE inhibitors) and angiotensin II receptor blockers (ARBs) to liver damage. Following ACEIs/ARBs, high levels of liver enzymes have been identified in hospitalized patients [89].
16. Despite new therapeutic strategies in the treatment of COVID-19, pirfenidone has not yet been tested in clinical trials. However, it has been reported that, due to the low side effects and potential benefits of pirfenidone, this drug can be considered a treatment target [109].

Pirfenidone has been proposed as an antifibrotic treatment in patients with idiopathic pulmonary fibrosis (IPF). Furthermore, although this medication helps suppress liver fibrosis, it also increases the potential for liver disease. The reason for this liver damage is unclear, but an elevation in liver enzyme levels has been documented in these patients [100].

Finally, since the SARS-CoV-2 infection causes symptoms such as fever and pain, it has been recommended that nonsteroidal anti-inflammatory medications and acetaminophen be used to alleviate symptoms. Overuse of acetaminophen (paracetamol), on the other hand, can cause liver problems [99].

It is worth noting that liver dysfunction caused by SARS-CoV-2 infection can result in improper drug metabolism in the liver, leading to increased liver toxicity.

## 10. Clinical Approach in Liver Injury and COVID-19

As mentioned, the experiments have found that the levels of AST, ALT, ALP, gamma-glutamyl transferase (GGT), and bilirubin in the deceased COVID-19 patients are considerably higher than those in in the recovering patients [12]. GGT as a diagnostic marker for cholangiocyte damage is elevated by more than 72% in extreme COVID-19 patients [45]. Furthermore, peripheral blood testing revealed a substantial decline in CD4 and CD8 T cells, an improvement in CC chemokine receptor (CCR) 6+ *T* helper (Th) 17 CD4 T cells, and CD8 cell granulation, both of which are associated with liver manifestations. It has also been reported that, despite significant reductions in CD4 and CD8 cell counts, they are over-reactive [11]. During a study, it was found that the rate of AST/ALT in patients with mild symptoms was 18.2/19.8% and in patients with severe symptoms was 39.4/28.1% [19].

Furthermore, when compared to normal levels, AST and ALT increase one- to two-fold, protecting the liver from ischemic or hypoxic damage [56]. It was also discovered that people using lopinavir/ritonavir had a 56.1% rise in liver enzymes compared to individuals without treatment. A rise in liver enzymes caused by liver injury or the use of antiviral medications, on the other hand, is controversial. Moreover, in such a way that the patients recovered and died, respectively, AST levels of more than 40 U/L were observed in 16% and 52% of subjects, and ALT levels were observed in 27% and 19% of subjects. Hypoalbuminemia was also reported in 65% of deaths and 14% of recovery cases [12].

## 11. COVID-19 Influence on Liver Cancer Management

Health care systems are facing unprecedented challenges as a consequence of the coronavirus disease 2019 (COVID-19) pandemic, which has a particularly adverse effect on individuals with liver cancer (LC) [110]. According to reports, the initial wave of the COVID-19 pandemic had a significant impact on the normal management of patients with liver cancer [110]. Several organizations offered several advice based on expert opinion data at the start of the first wave of the COVID-19 pandemic to lessen its effects on LC [110–112]. Given the extent of the disruption in patient care, which includes screening to diagnosis, staging, and treatment, the research's findings highlight the potential clinical significance of the changes that have been made [110]. They also predict that the COVID-19 pandemic will likely have a significant impact on outcomes [112]. Unfortunately, the crisis in health care leads to disruption in screening programs and subsequently increases the possible consequence of changing to a more advanced stage of diagnosis. Also, the progression, spread, and finally prognosis of the tumor can be affected by the delay in intervention measures such as transplantation, removal, or erosion. As a result, ultrasound screening delays due to the COVID-19 pandemic can be considered [110]. In general, the recovery status of patients and public health policy lead to a significant impact on LC management changes. The prospective analyses of project CERO-19 will give important information on the therapeutic efficacy of approaches used during these major health crises [110].

## 12. Conclusion

Due to the prevalence of COVID-19 and the severity of the clinical symptoms due to viral infection, the involvement of other organs such as the liver, heart, brain, and lungs during this pandemic increased disorders and severe complications of the disease. In some cases, an increase in mortality has been observed. As mentioned in the text, the mortality rate during this pandemic is primarily related to liver disorders and diseases. Also, this study explains the effect of SARS-CoV-2 and the number of diagnostic markers on disease outcomes. Besides, due to the presence of viral receptors on the epithelial cells of the small intestine, followed by the presence of viral RNA in fecal samples, as well as the possibility of virus transmission from the intestine through the bloodstream to the liver, measures to prevent fecal-oral transmission of the virus are essential.

Furthermore, according to a study by Chen et al., 5% of people with COVID-19 had chronic liver disease, of which about 76.9% died. As a result, they indicate the importance of liver dysfunction in patients with SARS-CoV-2 [12]. Also, Chew et al. find that, in addition to the virus's direct influence on liver damage, variables including ischemia and drug-induced liver damage play a substantial role in liver damage during SARS-CoV-2 infection. However, this liver damage does not always result in liver failure and death [88]. Therefore, monitoring and providing preventive measures to inhibit and control infection in high-risk patients and those susceptible to severe COVID-19 are essential, such as in people with pre-existing liver disease, HCC, liver transplant patients, and patients taking antiviral drugs. Furthermore, the mortality rate is higher in the elderly and in people with pre-existing diseases. However, information on the effect of pre-existing chronic liver disease on the severity of COVID-19, as well as the effect of this disease on liver function, is insufficient and requires more extensive and detailed research.

## Figures and Tables

**Table 1:** The role of inhibitory medications in liver transplantation patients.

Medication	Function
Calcineurin inhibitors	The findings show that calcineurin inhibitors have antiviral effects by reducing the cytokine cascade during COVID-19 disease [80, 81].
mTOR inhibitors	On the other hand, the mTOR inhibitors have a beneficial impact on memory T cell function and inhibit the replication of viruses such as cytomegalovirus, herpes virus-8, Epstein-Barr, and human immunodeficiency virus [82].
Mycophenolate	According to studies, mycophenolate also has a cytostatic impact on activated lymphocytes [83]. Also, it causes an exacerbation of SARS-CoV-2 virus infection [84].

**Table 2:** Clinical symptoms and functions of drug-induced liver injury.

Drug	Clinical symptoms	Functions
Remdesivir	Elevated liver enzymes [92]	Hepatic dysfunction due to an association with P-GP inhibitors [93]
Methylprednisolone	Not reported	Modulation of cytokine cascades [52]

Tocilizumab	Elevates the serum ALT [26]	Alleviate inflammatory cytokines
		Acute liver disease
		Acute hepatitis liver transplantation in some cases [93]
Hydroxychloroquine	Not reported	Relieve inflammation caused by the disease [94–96]

Azithromycin	Hepatocellular [97]	(1) Induce acute liver injury within a few days of beginning therapy
		(2) Clinical signs of cholestatic hepatitis within 1 to 3 weeks of starting treatment [97, 98]
Interferon-beta	Elevated the ALT/AST/ALP serum [98]	Leads to asymptomatic and minor liver disorders [98]
Lopinavir	Significantly high level of serum's aminotransferase [98]	Protease inhibitor [98]
Ritonavir	Causes self-limiting and asymptomatic elevated in liver enzymes [98]	Improve lopinavir levels by inhibiting the liver enzyme CYP450; as a consequence, CPY3A4 produces a toxic mediator of ritonavir or other metabolized factors, which causes liver disorders [94–96]
Baricitinib	Temporary elevate in transaminase enzyme levels [98, 99]	Inhibitor of JAK-1/2 [98, 99]
Oseltamivir	There is no enough detail about its	Effect on liver function [12]
Ribavirin	Extreme hemolysis [12]	Broad spectrum [12]
ACE inhibitors and ARBs	Promotes the levels of liver enzymes [89]	Not reported

Pirfenidone	Promotes the levels of liver enzymes [100]	(1) Suppress liver fibrosis
		(2) Increase the potential of liver disease [100]

mTOR inhibitors: mammalian target of rapamycin, P-GP: P-glycoprotein, ALT: alanine aminotransferase, ALT: alanine aminotransferase, ALP: alkaline phosphatase, CYP450: cytochrome P450, CPY3A4: cytochrome P450 3A4, JAK-1/2: Janus kinase 1/2, ACE: angiotensin-converting enzyme, and ARBs: angiotensin II receptor blockers.

## Data Availability

The datasets generated during the current study are available from the corresponding author on reasonable request.

## Additional Points

This manuscript is a review type and have not require to registration number.
